# Low expression of centrosomal protein 78 (CEP78) is associated with poor prognosis of colorectal cancer patients

**DOI:** 10.1186/s40880-016-0121-3

**Published:** 2016-06-29

**Authors:** Meifang Zhang, Tingmei Duan, Li Wang, Jianjun Tang, Rongzhen Luo, Ruhua Zhang, Tiebang Kang

**Affiliations:** State Key Laboratory of Oncology in South China, Collaborative Innovation Center for Cancer Medicine, Sun Yat-sen University Cancer Center, Guangzhou, 510060 Guangdong P. R. China; Department of Pathology, Sun Yat-sen University Cancer Center, Guangzhou, 510060 Guangdong P. R. China; Research Department, Sun Yat-sen University Cancer Center, Guangzhou, 510060 Guangdong P. R. China

**Keywords:** Colorectal cancer, CEP78, Cell cycle, Prognosis

## Abstract

**Background:**

Centrosomal protein 78 (CEP78) has been characterized as a component of the centrosome required for the regulation of centrosome-related events during the cell cycle, but its role in human cancers remains unclear. This study aimed to investigate the role and the clinical value of CEP78 in colorectal cancer (CRC).

**Methods:**

Quantitative real-time polymerase chain reaction (qRT-PCR) and immunohistochemistry were performed to examine CEP78 expression in CRC tissues and adjacent noncancerous tissues. The association between CEP78 expression and clinical outcomes of CRC patients was determined. The effect of CEP78 on cell growth was examined in vitro by 3-(4,5-dimethyl-2-thiazolyl)-2,5-diphenyl-2-H-tetrazolium bromide (MTT) assay, colony formation, and flow cytometry assays and in vivo using a nude mouse model.

**Results:**

The expression level of CEP78 was significantly lower in tumor tissues than in the adjacent normal tissues (*P* < 0.01). Low CEP78 expression was significantly associated with poor differentiation (*P* = 0.003), large tumor size (*P* = 0.017), lymphatic metastasis (*P* = 0.034), distant metastasis (*P* = 0.029), and advanced stage (*P* = 0.011). Kaplan–Meier analysis indicated that patients with low CEP78 expression had shorter survival than those with high CEP78 expression (*P* < 0.01). Overexpression of CEP78 in CRC cells significantly reduced cell viability and colony formation in vitro and halted tumor growth in vivo. Further study showed that CEP78 reintroduction in CRC cells resulted in G_2_/M phase arrest rather than cell apoptosis.

**Conclusions:**

CEP78 might function as a tumor suppressor and serve as a novel prognostic marker in CRC.

## Background

Colorectal cancer (CRC) is the third most prevalent cancer worldwide, with over 1.4 million new cancer cases diagnosed and 693,900 deaths in 2012 [1]. Additionally, the incidence and mortality of CRC are increasing yearly in China [2]. The development of CRC is a long and complicated process accompanied by the combined activation of oncogenes and inactivation of tumor suppressor genes [3, 4]. Although an increasing number of molecules have been found to be implicated in CRC over the years, it is still necessary and urgent to identify genes that are crucial for tumor development in specific genetic contexts, which could further improve the early detection, prevention, intervention, and prognostic evaluation of patients with CRC.

The centrosome is a critical cellular organelle that functions as the microtubule-organizing center of cells and is critically involved in cell division [3, 5]. Centrosomal proteins (CEPs) are usually defined as the molecules that are localized at the centrosome and participate in the regulation of centrosome-related function [6]. For instance, CEP76 specifically prevents centriole re-duplication by limiting duplication to one per cell cycle [7]. CEP120 is required for centriole duplication, maturation, and subsequent ciliogenesis, which physiologically affects cerebellar and embryonic development [8]. Regarding the cell cycle, centrosome duplication is periodically regulated in a highly choreographed manner and is accordingly coupled to activities of cyclin-dependent kinases (CDKs). Reciprocally, the activities of CDKs are affected by centrosome status. The inhibition or silencing of several centrosome-associated proteins, such as dynactin, poly(ADP-ribose) polymerase 3 (PARP3), centriolin, and A-kinase anchor protein 450 (AKAP450), could block cell cycle progression [9]. Because dysregulation of the cell cycle commonly occurs during tumor development [10], CEPs potentially participate in tumorigenesis under certain genetic contexts. Recently, CEP55 was found to be overexpressed and positively correlated with tumor growth in CRC [11, 12]. However, the functions of CEPs in tumor development remain elusive and merit further investigation.

The *CEP78* gene, located on chromosome 9q21.2, encodes a 78-kDa protein CEP78. CEP78 was identified as a component of the centrosome through mass spectrometry-based proteomic analysis. However, whether and how CEP78 is coordinated with centrosome activities remain unknown [6]. A previous study demonstrated that CEP78 might be involved in treatment-associated immune responses in patients with prostate cancer [13]. However, the clinical implication and functions of CEP78 relevant to tumorigenesis are largely unknown.

In this study, the expression of CEP78 in CRC was determined. Additionally, the relationship between the clinicopathologic parameters of CRC patients and CEP78 was examined. The antitumor effect of CEP78 was investigated in vitro and in vivo.

## Methods

### Cell lines and patient tissue samples

Paraffin-embedded primary specimens were obtained from 237 CRC patients with complete clinicopathologic data. Matched adjacent normal tissues were obtained from 158 patients. The patients were diagnosed at the Sun Yat-sen University Cancer Center (SYSUCC), Guangzhou, China between 1999 and 2007. None of the patients had received radiotherapy or chemotherapy prior to surgery. The cohort consisted of 127 (53.6%) men and 110 (46.4%) women. The clinical stage of CRC was evaluated on the basis of the TNM classification system [14]. The clinicopathologic characteristics of these CRC patients are shown in Table 1. An additional eight paired fresh CRC tissues, along with the corresponding non-tumorous tissues, were collected for RNA extraction. All samples were anonymous. Written consent was obtained from all patients. The study and consent procedure were approved by the Institutional Research Ethics Committee of SYSUCC.

**Table 1:** Association between clinicopathologic variables of colorectal cancer patients and centrosomal protein 78 (CEP78) expression

Variable	Total (cases)	CEP78 expression [cases (%)]		*P* value
		Low	High	
Age				0.881
≤ 50 years	88	64 (72.7)	24 (27.3)	
> 50 years	149	102 (68.5)	47 (31.5)	
Gender				0.102
Female	110	82 (74.5)	28 (25.5)	
Male	127	84 (66.1)	43 (33.9)	
Size				0.017
≤4 cm	68	40 (58.8)	28 (41.2)	
>4 cm	169	126 (74.6)	43 (25.4)	
Differentiation				0.003
Well	7	3 (42.9)	4 (57.1)	
Moderate	171	112 (65.5)	59 (34.5)	
Poor	59	51 (86.4)	8 (13.6)	
Depth of tumor				0.060
T1 + T2	44	26 (59.1)	18 (40.9)	
T3 + T4	193	140 (72.5)	53 (27.5)	
Lymphatic metastasis				0.034
Absent	112	71 (63.4)	41 (36.6)	
Present	125	95 (76.0)	30 (24.0)	
Distant metastasis				0.029
Absent	178	118 (66.3)	60 (33.7)	
Present	59	48 (81.4)	11 (18.6)	
Stage				0.011
I + II	91	55 (60.4)	36 (39.6)	
III + IV	146	111 (76.0)	35 (24.0)	

CRC cell lines (SW480, SW620, HCT116, HT29, DLD1, LOVO, RKO, and THC8307) were obtained from the American Type Culture Collection (ATCC) and cultured according to the instructions of ATCC.

### Construction of stable cell lines overexpressing CEP78

Full-length human CEP78 cDNA was cloned into a pSin-puro vector (GenePharma, Shanghai, China), and CEP78 was verified by DNA sequencing. The primers were 5′-GGAATTCCATATGACCATGATCGACTCCGTGAAGCTG-3′ (forward) and 5′-CTAGCTAGCTCAGGAATGCAGGTCCTTTCC-3′ (reverse). pSin-puro-CEP78 or the pSin-puro empty vector was co-transfected with pMD.2G and psPAX2 into HEK-293T cells for 48 h. The recombinant viruses were collected and used to infect HT29 and HCT116 cells, which were cultured with 8 μg/mL polybrene for 24 h. Stable lines were selected with 1 μg/mL of puromycin for 2 weeks.

### RNA extraction and quantitative real-time polymerase chain reaction (qRT-PCR)

The total RNA of fresh CRC specimens was isolated using Trizol (Invitrogen, Carlsbad, CA, USA) according to the manufacturer’s protocol. First-strand cDNA was synthesized using PrimeScript^®^ RT reagent kit with gDNA Eraser (Fermentas, Burlington, ON, Canada). Semi-qRT-PCR and qRT-PCR were performed for the detection of *CEP78* mRNA using Advantage HD DNA Polymerase Mix (Takara, Shimogyo-ku, Kyoto, Japan) and SYBR^®^ Premix Ex Taq™ II (Takara), respectively. The primer sequences were as follows: 5′-TGGCAGGGAGCAGATCACA-3′ (forward) and 5′-AAGCCAGCCATACAGTCAAGA-3′ (reverse) for *CEP78*; 5′-ACAGTCAGCCGCATCTTCTT-3′ (forward) and 5′-GACAAGCTTCCCGTTCTCAG-3′ (reverse) for *GAPDH*. The qRT-PCR reaction conditions were as follows: 98 °C for 2 min followed by 35 cycles of 98 °C for 10 s, 60 °C for 5 s, 72 °C for 30 s, and 72 °C for 10 min, and, finally, hold at 4 °C. The products were analyzed by 2% agarose gel electrophoresis and stained with ethidium bromide for visualization using ultraviolet light.

### Western blotting

Western blotting was performed as previously described [15]. Cell lysates were resolved by sodium dodecyl sulfate-polyacrylamide gel electrophoresis (SDS-PAGE) and transferred to polyvinylidene fluoride (PVDF) membranes, which were then incubated with anti-CEP78 antibody (Sigma, St. Louis, MO, USA) in 5% non-fat milk. Then, the membrane was incubated with horseradish peroxidase (HRP)-conjugated anti-rabbit secondary antibody. The blots were then visualized using an electrochemiluminescence (ECL) kit (Beyotime, Nangtong, Jiangsu, China).

### MTT assay

The 3-(4,5-dimethylthiazol-2-yl)-2,5-diphenyltetrazolium bromide (MTT) assay was used to measure the cell viability as previously described [16]. Cells were seeded at a density of 2500 or 3000 per well in 96-well microplates. The cells were incubated with MTT for 4 h, the optical density (OD) was detected at 490 nm with a microplate reader, and measurements were acquired once per day for 5 days. The results are presented as the mean ± standard error of mean (SEM) of three independent experiments.

### Colony formation assay

Cells were plated in the 6-well culture plates at 250 cells per well, and each group had 3 wells. Cells were washed twice with phosphate-buffered saline (PBS) after incubation for 15 days at 37 °C, and then stained with Giemsa solution. The number of colonies containing ≥50 cells was counted under a light microscope (Olympus, Orinpasu Kabushiki-gaisha, Japan).

### Immunohistochemistry (IHC)

IHC was performed as previously described [17]. Sections of paraffin-embedded specimens (4 μm in thickness) were baked and deparaffinized. Sections were stained with an anti-CEP78 antibody (Sigma Aldrich), and the antigen was detected using a secondary anti-rabbit HRP-conjugated antibody. Subsequently, the sections were stained with 3,3′diaminobenzidine (DAB) and counterstained with hematoxylin. CEP78 expression was evaluated by two independent pathologists. The IHC staining score was calculated. Using the H-score method, we multiplied the percentage score by the staining intensity score. The percentage of positively stained cells was scored as 0 (0%), 1 (1%–25%), 2 (26%–50 %), 3 (51%–75%), or 4 (76%–100%). Staining intensity was scored as 0 (negative staining), 1 (weak staining), 2 (moderate staining), or 3 (strong staining). The median score was chosen as the cut-off value to define low and high CEP78 expression [16].

### Apoptosis assays

The effect of CEP78 on apoptosis was examined. HT29 and HCT116 cells were transfected with a CEP78 overexpression vector for 48 h. Cells were then collected by centrifugation at 1000 r/min for 10 min and treated with Annexin V-FITC and propidium iodide (Roche, New York, NY, USA) for 15 min. The cell suspension was immediately analyzed by flow cytometry (Beckman-Coulter, Inc, Fullerton, CA, USA) to evaluate cell apoptosis. At least 10,000 events were collected for each sample.

### Animal experiments

Twenty-four male BALB/c athymic nude mice (4 weeks old) were obtained from Guangdong Medical Laboratory Animal Center (Guangzhou, China). Mice were randomly separated into the empty vector group and the CEP78 group with inoculation of HCT116 or HT29 cells. Each group included 6 mice. All animal experiments were performed in the animal institute of SYSUCC according to the principles and procedures approved by the Medical Experimental Animal Care Commission of SYSUCC. To assess CRC tumor growth in vivo, 1 × 10^6^ HCT116 or HT29 cells were injected subcutaneously into the dorsal flank of each mouse. Tumor size was measured every 2 days. After 20 days, the mice were euthanized, and the xenografts were weighed.

### Statistical analysis

SPSS software (version 16.0, Chicago, IL, USA) was used for statistical analysis. The relationship between CEP78 expression and the clinicopathologic parameters was examined by Student’s *t* test (Fisher’s exact test was chosen when the minimum expected cell count was less than 5). Overall survival (OS) curves were plotted by the Kaplan–Meier method and analyzed using the log-rank test. Univariate and multivariate Cox regression analyses were used to evaluate survival data. Differences were considered statistically significant when *P* values were less than 0.05 (two tailed).

## Results

### The expression level of CEP78 was decreased in CRC tissues

**Fig. 1:**
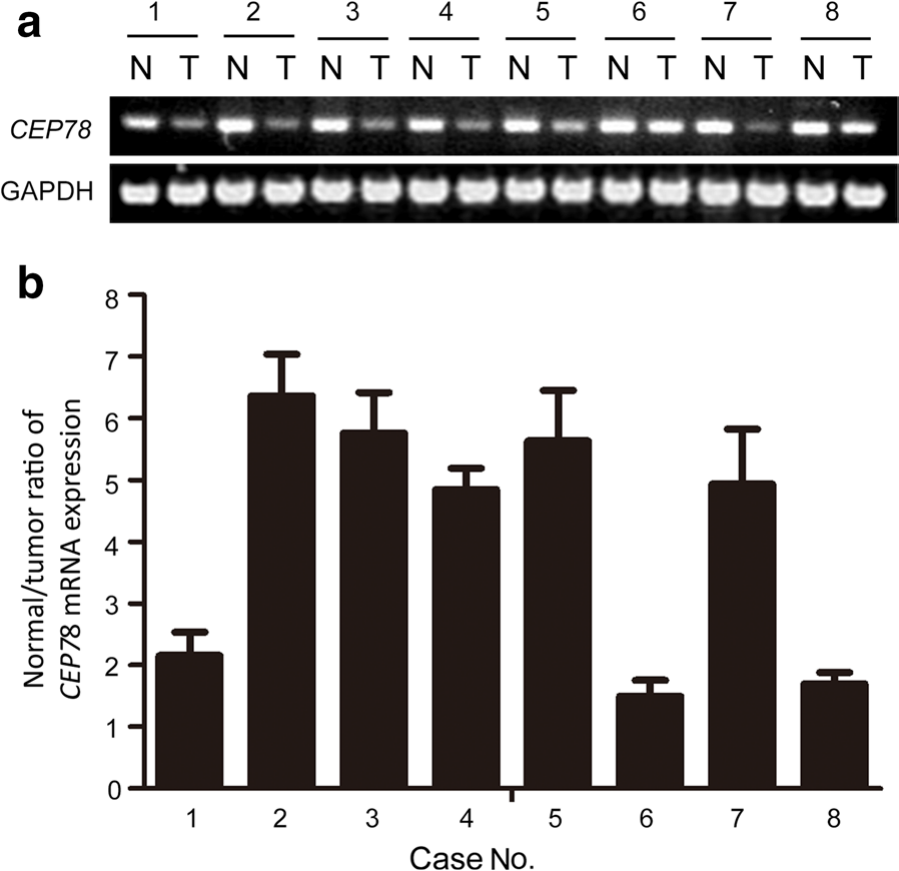
The mRNA level of centrosomal protein 78 (*CEP78*) in 8 paired colorectal cancer (CRC) tissues and non-tumorous tissues. **a** *CEP78* mRNA levels in CRC tumor tissues (“T” on this* panel*) and the non-tumorous counterparts (“N” on this* panel*) were determined. **b** quantitative real-time polymerase chain reaction (qRT-PCR) was used to examine relative *CEP78* mRNA expression. The normal/cancer ratio is shown

We detected the mRNA levels of *CEP78* in CRC tissues and normal tissues by qRT-PCR. As shown in Fig. 1, *CEP78* mRNA levels were significantly lower in most tumor tissues (5 out of 8) than in normal tissues.

**Fig. 2:**
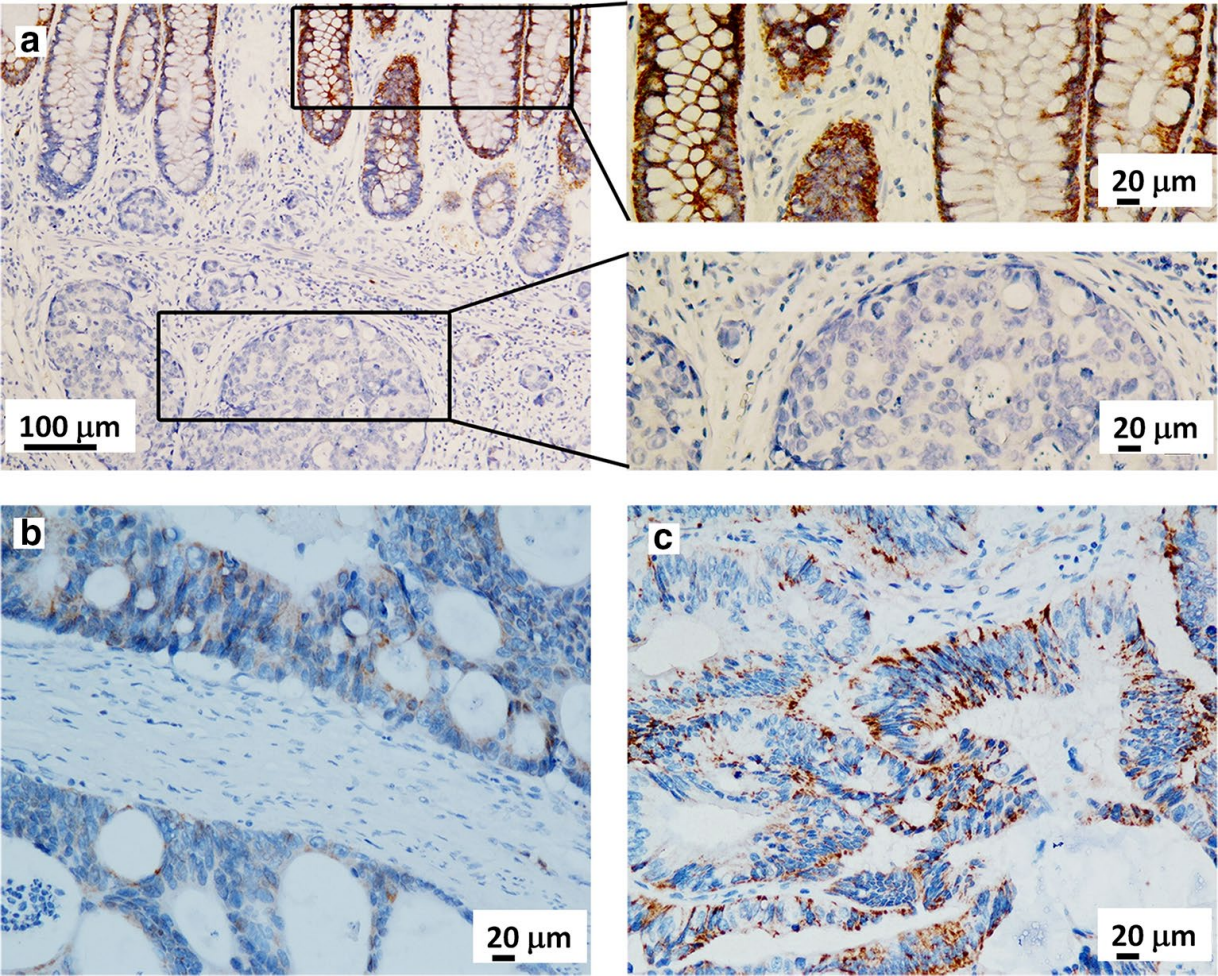
Expression of CEP78 in CRC tissues is determined by immunohistochemistry (IHC). CEP78 is found in the cytoplasm of tumor cells and adjacent normal cells. **a** Representative image of CRC tissues with no expression of CEP78 and the adjacent normal tissues with strong expression. **b** Representative image of CRC tissues with weak expression of CEP78. **c** Representative image of CRC tissues with strong expression of CEP78

To determine the potential relationship between CEP78 expression and CRC development, IHC analyses were performed to evaluate CEP78 protein levels in 237 CRC samples. Overall, CEP78 was found to be mainly distributed in the cytoplasm of tumor and non-tumor cells (Fig. 2). According to the median score (5.85) of CEP78 staining, tissue samples were categorized into low and high CEP78 expression groups. Surprisingly, we found that high CEP78 expression was detected in 30.0% (71/237) of samples of tumor tissues and 89.2% (141/158) of samples of adjacent normal tissues (*P* < 0.001). These results indicated that CEP78 expression was significantly suppressed in tumor tissues during CRC development.

### Association between CEP78 expression and clinicopathologic factors

Clinicopathologic factors were further analyzed between the high and low CEP78 expression groups. As shown in Table 1, CRC patients with low CEP78 expression had a higher tendency to exhibit poor differentiation (*P* = 0.003), large tumor size (*P* = 0.017), lymphatic metastasis (*P* = 0.036), distant metastasis (*P* = 0.019), and advanced stage (*P* = 0.008). However, we did not find significant associations between CEP78 expression and other clinicopathologic parameters, such as age, gender, and depth of tumor (*P* > 0.05).

### Low expression of CEP78 was associated with poor prognosis in CRC patients

**Fig. 3:**
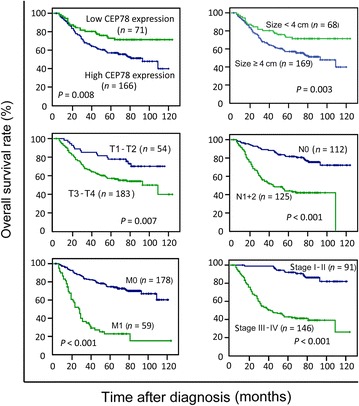
CEP78 expression is associated with overall survival of CRC patients. Patients with low CEP78 expression show a significantly poorer prognosis than those with high CEP78 expression. The associations between poor prognosis and large tumor size, deep tumor invasion, lymphatic metastasis, distant metastasis, and advanced tumor stage were also examined. *P* value was calculated by the log-rank test

To test the prognostic value of CEP78 in CRC patients, Kaplan–Meier survival analyses were conducted. In our cohort, 97 patients died of CRC. The median OS was 30.8 months. The 1-, 3-, and 5-year OS rates were 99.9%, 70.7%, and 60.2%, respectively. Of these 97 patients, 77 (79.4%) had low CEP78 expression, whereas 20 (20.6%) had high CEP78 expression. Kaplan–Meier survival analyses indicated that low CEP78 expression was associated with a poor prognosis (*P* = 0.008); poor prognosis was also associated with large tumor size (*P* = 0.003), deep tumor invasion (*P* = 0.007), lymphatic metastasis (*P* < 0.001), distant metastasis (*P* < 0.001), and advanced stage (*P* < 0.001) (Fig. 3).

Univariate analyses were also performed to examine the association between various factors and survival in our selected samples. The results shown in Table 2 indicated a significant association between patient survival and CEP78 expression (*P* = 0.009), tumor size (*P* = 0.004), tumor depth (*P* < 0.001), lymphatic metastasis (*P* < 0.001), distant metastasis (*P* < 0.001), and tumor stage (*P* < 0.001). However, other clinicopathologic features, such as age, gender, and histologic grade, were not significant prognostic factors (*P* > 0.05). Moreover, multivariate analysis showed that tumor size, distant metastasis, and tumor stage (*P* < 0.05), but not CEP78 expression (*P* = 0.191), were independent prognostic indicators in patients with CRC (Table 2).

**Table 2:** Univariate and multivariate analyses of prognostic values of clinicopathologic features and CEP78 expression for overall survival of CRC patients

Variable	Univariate analysis		Multivariate analysis	
	HR (95 % CI)	*P* value	HR (95 % CI)	*P* value
Age (≤50 years vs. >50 years)	0.994 (0.658–1.501)	0.976		
Gender (female vs. male)	1.072 (0.719–1.597)	0.733		
Tumor size (≤4 cm vs. >4 cm)	2.125 (1.273–3.548)	0.004	1.873 (1.095–3.323)	0.022
Tumor differentiation (well vs. moderate vs. poor)	0.214 (0.029–1.574)	0.096		
Depth of tumor (T1 + T2 vs. T3 + T4)	4.881 (2.129–11.19)	<0.001	0.815 (0.450–1.477)	0.500
Lymphatic metastasis (absent vs. present)	3.714 (2.351–5.865)	<0.001	1.537 (0.830–2.847)	0.172
Distant metastasis (absent vs. present)	4.954 (3.283–7.475)	<0.001	2.744 (1.701–4.425)	<0.001
TNM stage (I + II vs. III + IV)	7.373 (4.003–13.58)	<0.001	3.642 (1.492–8.894)	0.005
CEP78 expression (low vs. high)	0.520 (0.318–0.852)	0.009	0.787 (0.475–1.305)	0.354

*HR* hazard ratio, *CI* confidence interval

### Overexpression of CEP78 inhibited CRC proliferation in vitro

**Fig. 4:**
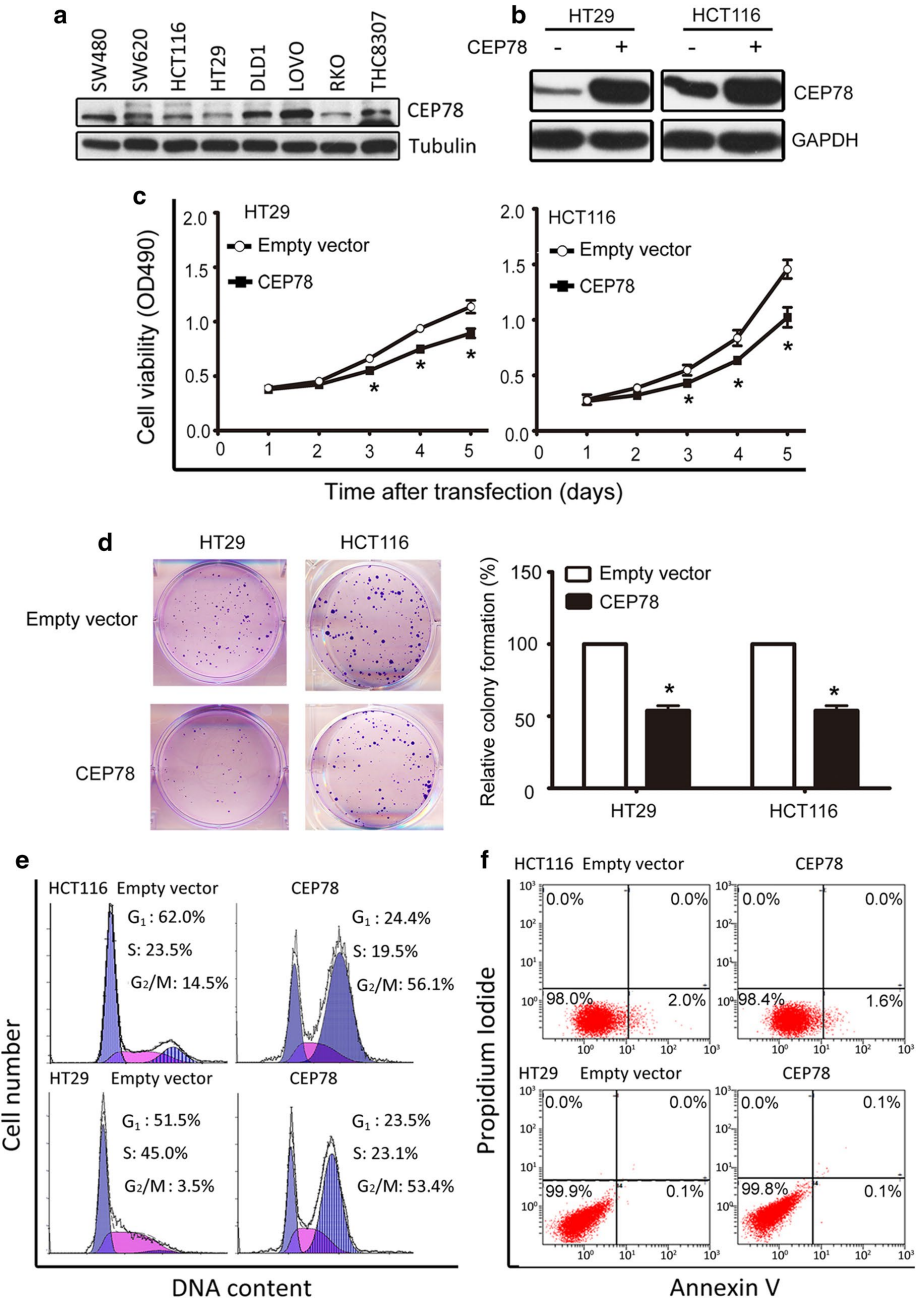
Overexpression of CEP78 in CRC cells inhibits cell viability in vitro. **a** Expression of CEP78 in CRC cell lines was determined by western blotting. **b** CEP78 protein levels were examined by western blotting in cells with or without CEP78 overexpression. **c** MTT assay results showed a significant reduction of viability in cells with CEP78 overexpression. **d** Colony formation was decreased in cells with CEP78 overexpression. The results of colony assay quantification are indicated as the mean ± standard error of the mean (SEM) of three independent experiments. *P < 0.05. **e** Cells were transfected with CEP78 or empty vector for 48 h. The distributions of cell cycle were determined by flow cytometry analyses. **f** Cells treated as described in e were subjected to apoptosis analyses, using Annexin V/PI staining

CEP78 was differently expressed in CRC cell lines (Fig. 4a). Two stable cell lines (HT29 and HCT116) overexpressing CEP78 were established (Fig. 4b). MTT assay analysis indicated that cell viability was dramatically impaired in cells overexpressing CEP78 (Fig. 4c). Consistently, the colony formation capability was also compromised in CEP78-overexpressing cells compared with the control cells (Fig. 4d). Further study showed that overexpression of CEP78 resulted in G_2_/M arrest. For instance, the percentage of HCT116 cells at G_2_/M phase increased from 14.5% in control cells to 56.1% in CEP78-overexpressing cells (Fig. 4e). Annexin V/PI assays revealed that CEP78 had no effect on cell apoptosis (Fig. 4f).

### Overexpression of CEP78 impaired the growth of CRC in vivo

**Fig. 5:**
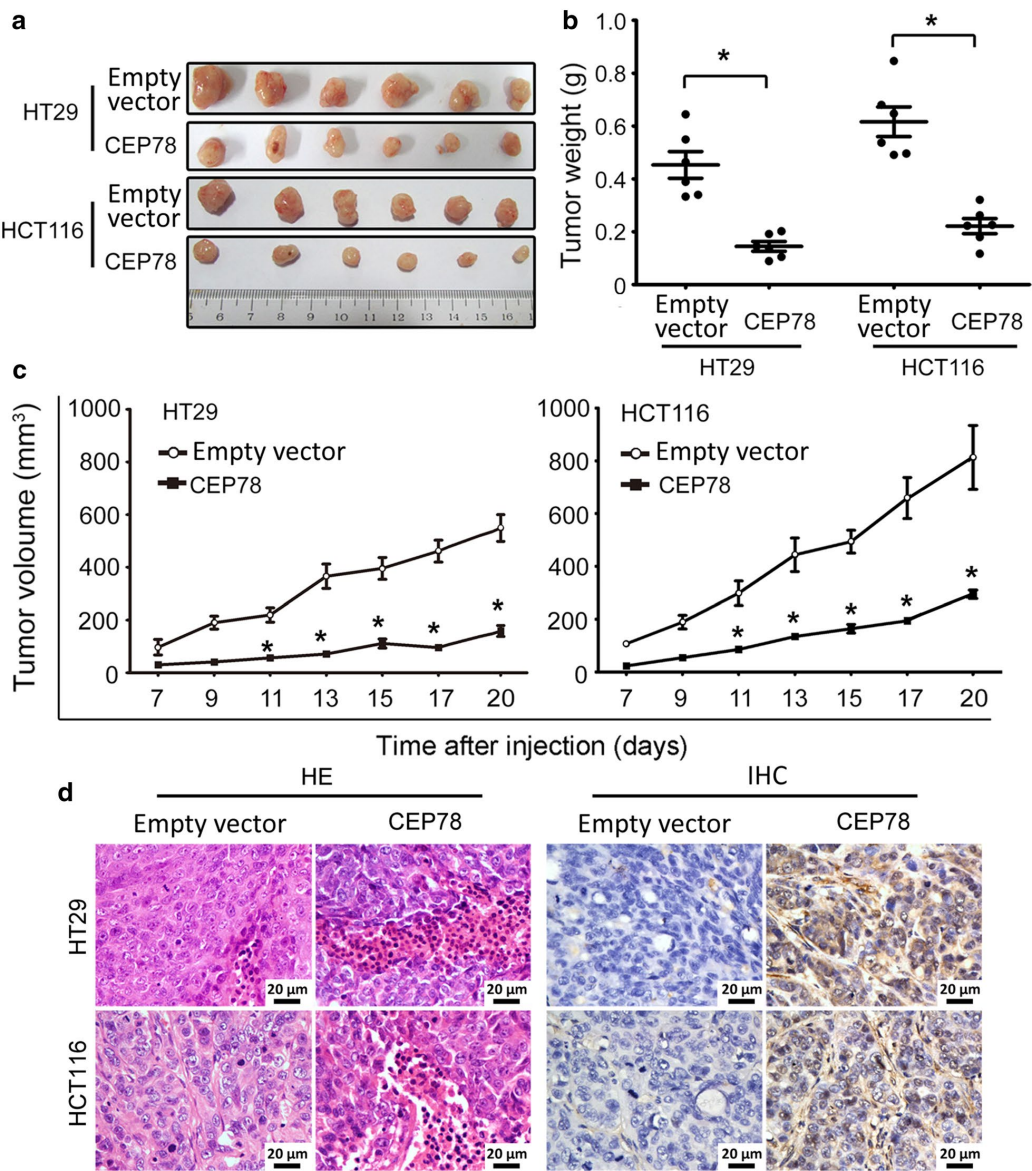
Overexpression of CEP78 in CRC cells impairs tumor growth in vivo. **a** Tumors were excised 20 days after injection. **b** The average weight of tumors from indicated cells in each group was assessed. **c** The average volume of tumors from indicated cells was measured every 2 days after injection. *P < 0.05. **d** The xenografts were sectioned and stained by hematoxylin and eosin (HE) or immunohistochemistry (IHC) with CEP78 antibody

To further characterize the function of CEP78 in CRC development, tumor cells with or without CEP78 overexpression were injected subcutaneously into nude mice, and both the weight and volume of tumors were measured 20 days after injection. The control groups showed rapid tumor growth. In sharp contrast, tumor growth was much slower in groups with CEP78 overexpression compared with the control groups, as indicated by the decreased weight and volume of tumors (Fig. 5a–c). The results of HE and IHC staining showed CEP78 overexpression in tumors formed by CRC cell lines (Fig. 5d).

## Discussion

Centrosome amplification, a hallmark of cancer, accounts for the unlimited proliferation of human cancers. Centrosome duplication is strictly controlled by a series of checkpoints in which CEPs play essential roles [6]. In this study, we showed that the expression of CEP78, a centrosome component, was markedly decreased in a large cohort of 237 patients with CRC. The silencing of CEP78 was closely associated with large tumor size, advanced tumor stage, lymphatic metastasis, and distant metastasis. Because patients with CRC often experience metastasis to the lymph nodes, liver, and lungs at late stages, CEP78 may serve as a predictor for tumor metastasis in CRC.

Patients with low CEP78 expression in our cohort were more likely to have shorter survival than those with high CEP78 expression. It has been well known that centrosome abnormality occurs in many types of cancer and is associated with poor outcomes [18]. Although no evidence indicates that CEP78 plays a role in centrosome duplication, one of its homologous proteins, CEP76, has been demonstrated to regulate the centrosome reduplication. Enforced CEP76 expression specifically inhibits centriole amplification but not normal centriole duplication [7], indicating that CEPs are capable of limiting centrosome copies. Therefore, we assume that lack of CEP78 results in centrosome amplification, and subsequently contributes to poor outcomes in CRC. Our data suggest that CEP78 may be of clinical significance, not only due to the large sample size in our study but also because CRC lacks common prognostic biomarkers.

CEPs are differentially expressed in human cancers. For example, CEP55 was demonstrated to be highly expressed in colon carcinoma [12], oral cavity squamous cell carcinoma [19], and bladder transitional cell carcinoma [20]. Reduction in CEP63 expression was found in bladder cancer [21]. Our data revealed that CEP78 expression was decreased in CRC. In vitro data showed that CEP78 overexpression significantly reduced cell viability and colony formation of CRC HT29 and HCT116 cells. The anti-tumor effect of CEP78 was further supported because CEP78 overexpression halted the growth of CRC xenografts. Mechanistically, CEP78 overexpression efficiently inhibited cell growth via arresting CRC cells at the G_2_/M phase. This is consistent with the roles of other CEPs in cell cycle regulation. Slaats et al. [22] showed that knockdown of CEP164 expression led to S phase arrest in renal cells. Ruiz-Miró et al. [23] reported that overexpression of CEP57 hindered NIH-3T3 cells at S phase. In our study, cell apoptosis was not observed in CRC cells with CEP78 overexpression. Collectively, our data provide evidence that CEP78 controls cell proliferation partly by modulating G_2_/M phase transition.

In summary, our data show that the expression of CEP78 is remarkably decreased in patients with CRC. Furthermore, the expression of CEP78 is robustly related to unfavorable clinical outcomes in patients with CRC. CEP78 overexpression suppressed cell growth via inducing G_2_/M phase arrest. This study therefore suggests CEP78 as a potential prognostic and therapeutic biomarker for CRC.
